# Novel Use for DOG1 in Discriminating Breast Invasive Carcinoma from Noninvasive Breast Lesions

**DOI:** 10.1155/2016/5628176

**Published:** 2016-03-02

**Authors:** Henghui Cheng, Shouhua Yang, Zhiling Qu, Sheng Zhou, Qiurong Ruan

**Affiliations:** ^1^Institute of Pathology, Tongji Hospital, Tongji Medical College, Huazhong University of Science and Technology, Wuhan 430030, China; ^2^Department of Obstetrics and Gynecology, Union Hospital, Tongji Medical College, Huazhong University of Science and Technology, Wuhan 430022, China

## Abstract

*Aims*. DOG1 has proven to be a useful marker of gastrointestinal stromal tumors (GISTs). Recently, DOG1 expression has also been reported in some non-GIST malignant tumors, but the details related to DOG1 expression in breast tissue remain unclear. The aim of this study was to detect the expression of DOG1 in the human breast and to evaluate the feasibility of using DOG1 to discriminate between invasive breast carcinoma and noninvasive breast lesions.* Methods and Results*. A total of 210 cases, including both invasive and noninvasive breast lesions, were collected to assess DOG1 expression immunohistochemically. DOG1 expression was consistently positive in breast myoepithelial cells (MECs), which was similar to the results obtained for three other MEC markers: calponin, smooth muscle myosin heavy chain (SMMHC), and P63 (*P* > 0.05 in all). Importantly, DOG1 was useful in discriminating invasive breast carcinoma from noninvasive breast lesions (*P* < 0.05).* Conclusions*. DOG1 is a useful marker of breast MECs, and adding DOG1 to the MEC identification panel will provide more sophisticated information when diagnosing uncertain cases in the breast.

## 1. Introduction 

DOG1, also known as TMEM16A, FLJ10261, ORAOV2, and anoctamin 1 [1–4], was identified as a typical finding on gastrointestinal stromal tumors (GISTs) using cDNA microarrays [1]. Through different functional assays, the corresponding protein has been identified as a calcium-regulated chloride channel protein (CaCC) with 8 transmembrane domains [2, 5, 6]. DOG1 has been shown to be sensitive and specific when detecting GISTs, although expression of DOG1 in other mesenchymal tumors, such as Ewing's sarcoma, angiosarcoma, leiomyosarcoma, and synovial sarcoma, has also been reported; there have also been occasional cases of DOG1 expression in malignant melanoma and germ cell tumors [1, 7–10]. Additionally, carcinomas of the liver, salivary glands, stomach, colon, esophagus, and lung have shown DOG1 immunoreactivity [9–12]. However, little is known about the clinical application of DOG1 expression beyond GIST.

In normal tissues, DOG1 has been described in a variety of epithelia, including the gastrointestinal tract, salivary gland, lung, pancreas, prostate, and kidney [13–15]. In the breast, both myoepithelial cells (MECs) and luminal epithelial cells have shown DOG1 immunoreactivity in a few cases [15], although no study has evaluated DOG1 expression in different types of breast lesions. Thus, the study of DOG1 in the breast remains far from complete.

In this study, we aimed to assess the expression and immunohistochemical orientation of DOG1 in various breast lesions and to evaluate the use of DOG1 as a novel myoepithelial marker for discriminating between invasive breast carcinoma and noninvasive breast lesions.

## 2. Materials and Methods

### 2.1. Patients and Samples

The study was approved by the Institutional Ethics Committee. In all, 210 formalin-fixed paraffin-embedded cases between 2001 and 2013 at the Institute of Pathology, Tongji Hospital, Tongji Medical College, Huazhong University of Science and Technology, China, were included in the study. All available slides for each case were examined, and histopathological subtyping was performed according to the 2012 WHO classification of tumors of the breast [16]. The cohort consisted of 10 normal breast tissues, 65 invasive carcinomas, 60 intraductal proliferative lesions, 10 lobular neoplasia, 30 intraductal papillary lesions, 20 benign epithelial proliferations, 10 fibroepithelial tumors, and 5 epithelial-myoepithelial lesions (Table 1). The patients were all female and had a mean age of 44.6 years (range, 21–75). All specimens were obtained by mastectomy or excisional biopsy; cases of needle biopsy were excluded from the study. The diagnoses of all patients were confirmed by two experienced breast pathologists. In confusing cases that were rather difficult to diagnose, more than one MEC immunohistochemical marker was used to support the corresponding diagnosis.

### 2.2. Immunohistochemistry

For immunohistochemical analyses, 4 *μ*m-thick sections were obtained and deparaffinized in xylene and then hydrated in a graded series of alcohol. All cases with available tissue were stained using the corresponding antibodies. Primary antibodies included DOG1 (clone SP31, Spring Bioscience, USA; dilution 1 : 100) [17], calponin (clone CALP, DAKO, Denmark; dilution 1 : 300), SMMHC (M3558, DAKO, Denmark; dilution 1 : 200), and P63 (clone 4A4, DAKO, Denmark; dilution 1 : 100). Staining was then performed using the Envision system for 30 min at room temperature. Finally, samples were incubated with diaminobenzidine peroxidase substrate to give a brown stain, counter-stained with hematoxylin, and mounted with coverslips.

### 2.3. Immunohistochemistry Scoring of DOG1

Stains were evaluated based on a number of parameters. Subcellular localization was classified as membranous, including the location and extent (i.e., apical-luminal, basolateral, and complete), cytoplasmic or other. Immunoreactivity for each antibody was assessed separately and classified as absent (0–5% cells staining), focal (>5%, ≤50% cells staining), or diffuse (>50% cells staining). The intensity of DOG1 was classified as follows: weak or no staining received a score of 1, moderate staining received a score of 2, and strong staining received a score of 3. The results were scored by multiplying the percentage of positive cells (*P*) by the intensity (*I*), according to the following formula: *Q* = *P* × *I*; maximum = 300.

### 2.4. Statistical Analysis

The data in a category are presented as the frequency and percentage. The chi-square test or Fisher's exact test was used to analyze the frequency of immunoreactivity for DOG1, calponin, SMMHC, and P63 among different groups. Statistical analysis was performed using SPSS 13.0 on a Windows computer; *P* < 0.05 was considered statistically significant.

## 3. Results

### 3.1. DOG1 Immunohistochemical Staining Profile in the Normal Breast and Different Breast Lesions

**Figure 1:**
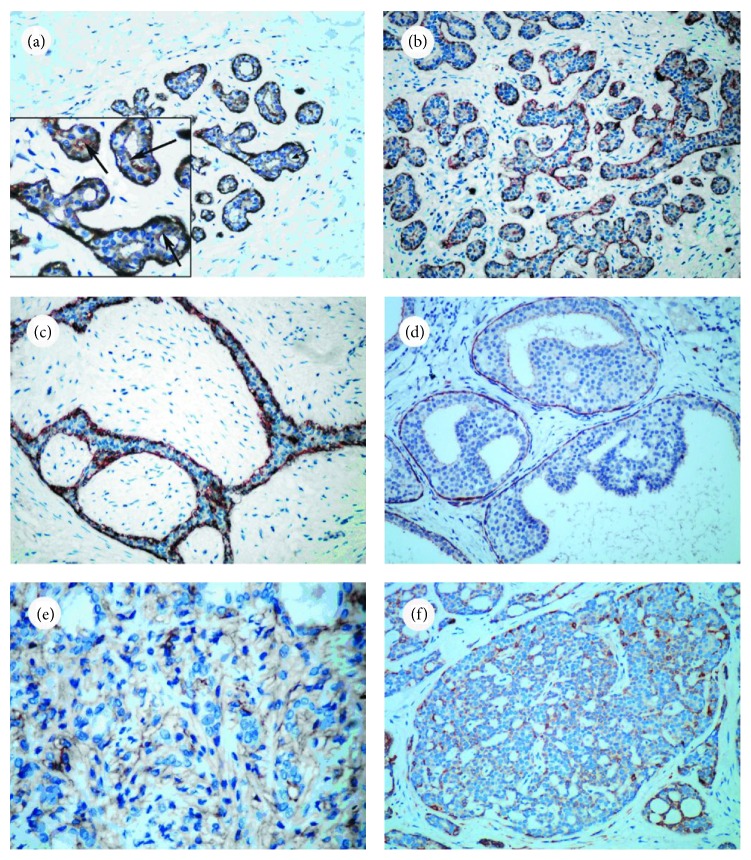
DOG1 expression in different breast lesions. (a) DOG1 was consistently expressed in normal duct and acini MECs (×200); the luminal epithelium showed randomly positive DOG1 expression in an apical-luminal membranous pattern with less intensity (arrow, ×400). Stromal cells were mostly negative. (b) DOG1 staining in MECs in adenosis (×200). (c) DOG1 was consistently positive in MECs in cases of fibroadenoma (×200). (d) DOG1 expression was positive in the duct in atypical ductal hyperplasia, but there was no DOG1 staining in atypical hyperplastic luminal cells (×200). (e) DOG1 staining in adenomyoepithelioma. There was negligible staining in both MECs and luminal epithelial cells (×400). (f) DOG1 staining in adenoid cystic carcinoma. There was negligible staining in MECs (×200).

Normal mammary gland tissue was present in 140 cases (130 para-lesion tissues and 10 normal breast cases). In each of these sections, DOG1 immunoreactivity was strongly and consistently positive in MECs of ducts and lobular acini (Figure 1(a)). The DOG1 staining pattern was cytoplasmic and complete, with occasional basolateral membranous staining. DOG1 was also randomly stained in luminal epithelial cells of ducts or acini, with cytoplasmic staining. However, unlike the expression pattern in MECs, DOG1 usually stained in an apical-luminal pattern in luminal epithelial cells (Figure 1(a)). Stromal cells of the breast were mostly unreactive in these cases.

In different breast lesions, DOG1 also showed MECs with membranous and cytoplasmic staining patterns. In usual ductal hyperplasia, intraductal papilloma, and fibroadenoma (Figure 1(c)), almost all MECs stained with DOG1. However, in other breast lesions, especially carcinoma in situ and intraductal papillary carcinoma, DOG1 staining in MECs was variable. There were 4 cases of ductal carcinoma in situ with no obvious DOG1 expression (Table 2). Compared to normal tissue, some cases showed focal DOG1 staining, and part of them showed a reduced intensity of staining. Meanwhile, calponin, SMMHC, and P63 also showed weak and variable staining or were absent in these ductal carcinoma in situ lesions (Table 2), and there were no significant differences between DOG1 and the other three markers (*P* > 0.05). In malignant breast tumors without MECs, no DOG1 staining was observed in MECs or neoplastic luminal epithelial cells. Additionally, the study included 5 cases of adenomyoepithelioma and 5 cases of adenoid cystic carcinoma; DOG1 staining was positive in 2 cases of adenomyoepithelioma and 1 case of adenoid cystic carcinoma (Figures 1(e) and 1(f)).

### 3.2. DOG1 as a Tool for Discriminating between Invasive Carcinoma and Adenosis or In Situ Carcinoma

**Figure 2:**
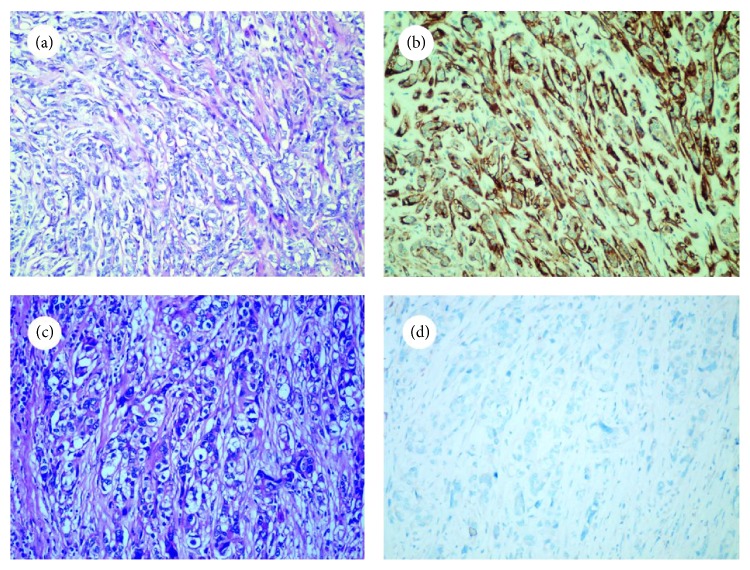
Comparison of DOG1 expression in adenosis and invasive carcinoma (×200). Hematoxylin and eosin staining of adenosis (a) and invasive carcinoma (c). It was difficult to distinguish one from the other based on morphology alone, although DOG1 staining showed the existence of MECs in adenosis (b) but not in invasive carcinoma (d).

There were 20 cases of adenosis, 50 cases of carcinoma in situ (40 cases of ductal carcinoma in situ and 10 cases of lobular carcinoma in situ), and 60 cases of invasive carcinoma (30 cases of invasive carcinoma of no special type, 10 cases of invasive lobular carcinoma, 10 cases of tubular carcinoma, and 10 cases of cribriform carcinoma). In each of these adenosis cases, DOG1 expression was detected in the outer layer of proliferated tubules (Figures 1(b) and 2(a)). However, there were 4 cases (20%) that showed only focal DOG1 staining (Table 2). The entire circumference of all the ducts and acini, corresponding to MECs, was also positive for calponin, SMMHC and P63 (Table 2).

**Figure 3:**
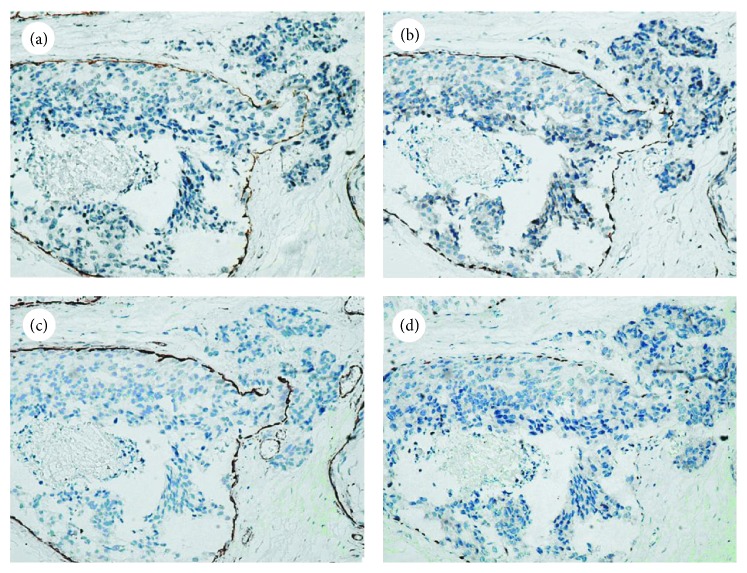
Comparison of different MEC markers in ductal carcinoma in situ with microinvasion (×200). (a) DOG1 expression was positive in MECs and negative in myofibroblastic cells and microinvasion lesions. (b) Calponin staining in MECs with partial stromal cell staining. (c) SMMHC staining in MECs, with obvious vessel staining. (d) P63 was strictly confined to the nuclei of MECs but showed discontinuous staining patterns in ductal carcinoma in situ with microinvasion compared to the other three markers.

Among the cases of carcinoma in situ, 46 of 50 (92%) showed DOG1 immunoreactivity in the outer duct layer (Figure 3(a)). There were 6 cases that showed discontinuous staining in a membranous pattern, especially in the foci of the intralobular extension of the neoplasm (Table 2). Only 4 cases of carcinoma in situ showed negligible DOG1 staining and were difficult to distinguish from invasive carcinoma (Table 2). The expression of calponin, SMMHC, and P63 was also decreased in these cases, and there were no obvious significant differences among the four markers (*P* > 0.05). However, compared to DOG1, calponin and SMMHC also decorated stromal myofibroblasts, vascular smooth muscle, and pericytes cells (Figures 3(b) and 3(c)), especially in stromal myofibroblasts lying just beneath the basal membrane, which might serve as a noninfiltration diagnostic trap. P63 proved to be extremely sensitive and specific in identifying MECs, but occasionally in cases of ductal in situ carcinoma with microinvasion, the appearance of P63 in a discontinuous staining pattern may morphologically suggest that MECs are absent (as shown in Figure 3(d)). These factors might somewhat reduce the overall utility of these markers in distinguishing between invasive and in situ lesions.

Compared to adenosis or carcinoma in situ, all cases of invasive carcinoma were DOG1 negative and lacked the myoepithelial component, which resulted in a significant difference between groups (Figure 5(a)). Other MEC markers were also absent in these sections, and there were no significant differences in occurrence between DOG1 and the other three markers (*P* > 0.05, see Table 2).

### 3.3. DOG1 as a Tool for Discriminating between Intraductal Papillary Carcinoma and Intraductal Papilloma

**Figure 4:**
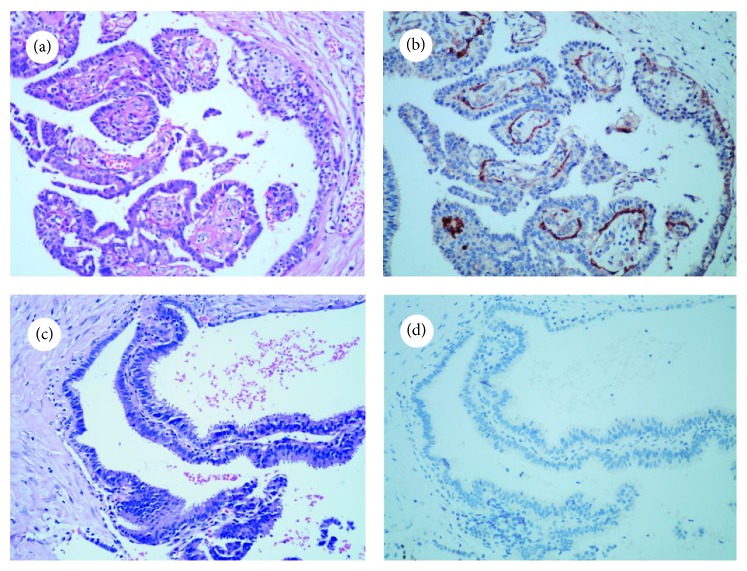
Comparison of DOG1 expression in intraductal papilloma and intraductal papillary carcinoma (×200). Hematoxylin and eosin staining of intraductal papilloma (a) and intraductal papillary carcinoma (c). The existence of MECs was clearly revealed by DOG1 staining in intraductal papilloma (b) but not in intraductal papillary carcinoma (d).

**Figure 5:**
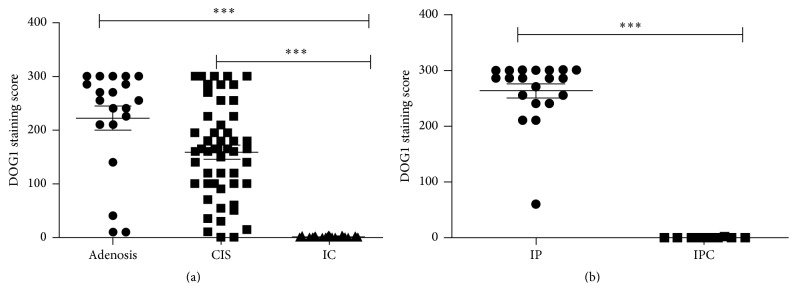
Comparison of DOG1 staining scores in unclear breast lesions. There were significant differences between adenosis and invasive carcinoma (*P* < 0.05); carcinoma in situ and invasive carcinoma (*P* < 0.05); and intraductal papilloma and intraductal papillary carcinoma (*P* < 0.05). CIS, carcinoma in situ; IC, invasive carcinoma; IP, intraductal papilloma; IPC, intraductal papillary carcinoma. (^*∗∗∗*^ *P* < 0.01).

Twenty cases of intraductal papilloma and 10 cases of intraductal papillary carcinoma were evaluated in this study. In all 20 cases of intraductal papilloma, DOG1 (Figure 4(b)) staining was positive in MECs (as previously shown), and in 1 case the staining was focal (Table 2). DOG1-positive MECs were absent in papillary projection areas in all 10 cases of intraductal papillary carcinoma, and there was a significant difference between intraductal papilloma and intraductal papillary carcinoma (Figure 5(b)). However, at the periphery of these papillary lesions in which MECs still existed, DOG1 staining became variable and was occasionally absent (Figure 4(d)).

## 4. Discussion

Histological morphology yields an accurate diagnosis in the vast majority of breast lesions. However, sometimes making a diagnosis can be problematic based on morphology alone, such as when distinguishing between benign and malignant tumors or in situ and invasive malignant lesions. Breast duct and acini are both composed of a double cell layer (inner luminal epithelial cells and outer MECs), and the presence of an intact peripheral MEC layer is a characteristic of all normal and benign breast lesions [18, 19]. Loss of the outer MEC layer is the hallmark of invasive breast carcinoma. Therefore, the identification of MECs is important for the differential diagnosis of breast lesions, and immunohistochemistry is an effective method for identifying these cells. Clinically, the most commonly used immunohistochemical markers are cytoplasmic antigens such as CD10, smooth muscle actin, muscle-specific actin, S-100 protein, calponin, smooth muscle myosin heavy chain (SMMHC) [18–21], and nuclear antigen P63 [21–23]. These antigens may also be present in stromal myofibroblast cells, vascular smooth muscle cells, and even luminal/epithelial cells [24, 25]. These caveats and exceptions, as well as related issues of marker specificity and sensitivity, highlight the diagnostic pitfalls that may be encountered when using myoepithelial markers to pathologically evaluate breast lesions. Currently, the combined use of more than one MEC marker is recommended to ensure accurate and reliable results.

In the current study, we demonstrated that DOG1 was expressed in breast tissue according to the staining results of 210 different breast tissues and lesions. We further revealed that DOG1 was consistently positive in MECs of both duct and lobular acini, with complete membranous staining. Sometimes the staining varied, especially in intralobular extensions of in situ carcinoma or retaining MECs at the circumference of the ducts invaded by tumor cells. Only a minor fraction of luminal and acinar epithelial cells showed apical-luminal immunoreactivity to DOG1, which limited the generation of noise when identifying MECs.

The expression of DOG1 in normal breast tissue has been reported previously. In previous research, both MECs and luminal epithelial cells showed positive DOG1 staining using different antibodies, such as K9, DOG1.1, or polyclonal antibody, but these cases were limited and a positive pattern was not clarified [1, 26]. Furthermore, the location of DOG1 in different breast lesions remained unknown, with only one study reporting that 9 of 11 (81.8%) cases of fibroadenoma showed positive DOG1 staining [27]. Although these findings showed DOG1 immunoreactivity in the breast, the specificity and significance of DOG1 staining needed to be demonstrated in more comprehensive breast studies. In our study, the expression of DOG1 in MECs and luminal epithelial cells was revealed both in normal breast tissue and a variety of breast lesions. Additionally, different staining patterns were also clarified. With findings supporting those of the present study, Chênevert et al. performed a comprehensive study of DOG1 expression in salivary tissues and found DOG1 immunoreactivity in both salivary serous acini and salivary tumors with intercalated duct differentiation; this result partly demonstrated the expression of DOG1 in salivary MECs [28]. In contrast to MECs in normal breast tissue, normal myoepithelial/basal cells of the salivary gland were uniformly DOG1 negative. This observation was also noted using the monoclonal antibody SP31 in our study (see Supplementary Figure 1 in Supplementary Material available online at http://dx.doi.org/10.1155/2016/5628176), which suggests that the expression of DOG1 in the myoepithelial/basal cells of these two organs may be the result of different mechanisms.

To demonstrate whether DOG1 could be a valuable marker of MECs in the breast, we compared DOG1 staining results from paraffin samples of unclear breast cases. Indeed, there were significant differences in DOG1 expression between invasive carcinoma and adenosis or in situ carcinoma (*P* < 0.05). In particular, DOG1 was effective at distinguishing adenosis or intralobular extension of in situ carcinoma from invasive carcinoma or microinvasion, similar to calponin, SMMHC, and P63 (Table 2, *P* > 0.05). There were also significant differences in DOG1 expression between intraductal papillary carcinoma and intraductal papilloma (*P* < 0.05). In fact, the combined assessment of calponin, SMMHC, and P63 could resolve most of the difficult cases, although there were still some cases in which MECs were difficult to delineate in all sections and for which additional MEC markers should be used. As shown in this study, DOG1 was both sensitive and specific in identifying MECs. Although occasional background staining of luminal epithelial cells occurred, this DOG1-positive population could be distinguished by morphological analysis. Thus, we believe that DOG1 may provide exact information about the presence or absence of the myoepithelial component as other three markers.

DOG1 is a calcium-dependent, receptor-activated chloride channel protein, indicating that it may serve different functions in different tissues [29–31]. Recently, new findings have suggested that DOG1 plays a potential exocrine/endocrine secretory role in pancreatic centroacinar cells and a subset of islet cells [12, 14]. In keeping with this secretory function, DOG1 has shown immunoreactivity in serous acini of the salivary gland. In this paper, we identified occasional DOG1 staining in normal luminal epithelial cells, especially in the apical-luminal pattern, which also confirms the secretory role of DOG1 in luminal cells. This DOG1 staining type decreased when luminal epithelial cells differentiated into tumor cells. Unlike the secretory role of DOG1 expression in luminal cells, the role of DOG1 in MECs is unclear and may offer new clues for understanding the function of DOG1. Because myofilaments in MECs have the main function of contraction, DOG1 may be involved in the contraction process by regulating cytosolic calcium as a transmembrane anion channel; however, this hypothesis needs to be studied further.

Additionally, in some epithelial-myoepithelial lesions, the myofilaments in MECs of these lesions are not always as well developed as in those lining the ducts of normal breast tissue, and the MECs markers could be undetected. In the current study, we assessed 5 cases of adenomyoepithelioma and 5 cases of adenoid cystic carcinoma and found that the sensitivity of DOG1 was apparently reduced in these cases. These types of “biphasic” tumors can also be encountered outside of the breast, and the immunoreactivity of DOG1 in these tumors varies in different locations. In the head and neck, DOG1 showed a 3/4 (75%) positive rate in cases of adenoid cystic carcinoma [27], and 9/24 (38%) showed diffuse staining in the salivary gland [28]. Additionally, the frequency of DOG1 staining declined when more solid area or high-grade transformation was seen in adenoid cystic carcinoma [28].

In summary, we show that DOG1 is consistently expressed in MECs of different breast tissues. In addition to occasional staining in luminal epithelial cells, there was no DOG1 immunoreactivity in stromal or vascular cells, which provides an advantage compared to other cytoplasmic antigens specific to MECs. Based on the ability of DOG1 to discriminate unclear breast cases, we propose that DOG1 is a reliable and specific MEC marker in formalin-fixed breast sections. Although in certain conditions DOG1 was not as accurate as other MEC markers, this protein can likely serve a candidate or supplementary role in the identification of MECs. Furthermore, our findings may promote further research into understanding the full function of DOG1.

## Supplementary Material

In this study, we detected the expression of DOG1 in normal salivary gland. The result showed that it was DOG1 positive in the apical-luminal surface of serous acini but uniformly DOG1 negative in myoepithelial/basal cells, which was different from the performance of DOG1 in normal breast tissue.

## Figures and Tables

**Table 1:** Normal tissue and different lesions included in this study.

Diagnosis	Number of cases
Normal	10
Invasive carcinoma of no special type	30
Invasive lobular carcinoma	10
Tubular carcinoma	10
Cribriform carcinoma	10
Adenoid cystic carcinoma	5
Usual ductal hyperplasia	10
Atypical ductal hyperplasia	10
Ductal carcinoma in situ	40
Lobular carcinoma in situ	10
Intraductal papilloma	20
Intraductal papillary carcinoma	10
Adenosis	20
Fibroadenoma	10
Adenomyoepithelioma	5

**Table 2:** Frequency of immunoreactivity for DOG1, calponin, SMMHC, and P63 in invasive and noninvasive breast lesions.

Cases	DOG-1			Calponin			SMMHC			P63			*P*
	− (%)	+ (%)	++ (%)	− (%)	+ (%)	++ (%)	− (%)	+ (%)	++ (%)	− (%)	+ (%)	++ (%)	
Adenosis (20 cases)	0/20	3/20	17/20	0/20	1/20	19/20	0/20	2/20	18/20	0/20	1/20	19/20	>0.05
	(0)	(15)	(85)	(0)	(5)	(95)	(0)	(10)	(90)	(0)	(5)	(95)	

CIS (50 cases)	4/50	6/50	40/50	2/50	6/50	42/50	1/50	6/50	43/50	0/50	4/50	46/50	>0.05
	(8)	(12)	(80)	(4)	(12)	(84)	(2)	(12)	(86)	(0)	(8)	(92)	

IC (60 cases)	60/60	0/60	0/60	60/60	0/60	0/60	60/60	0/60	0/60	59/60	1/60	0/60	>0.05
	(100)	(0)	(0)	(100)	(0)	(0)	(100)	(0)	(0)	(98.3)	(1.7)	(0)	

IP (20 cases)	0/20	1/20	19/20	0/20	2/20	18/20	0/20	1/20	19/20	0/20	0/20	20/20	>0.05
	(0)	(5)	(95)	(0)	(10)	(90)	(0)	(5)	(95)	(0)	(0)	(100)	

IPC (10 cases)	10/10	0/10	0/10	10/10	0/10	0/10	9/10	1/10	0/10	10/10	0/10	0/10	>0.05
	(100)	(0)	(0)	(100)	(0)	(0)	(90)	(10)	(0)	(100)	(0)	(0)	

CIS, carcinoma in situ; IC, invasive carcinoma; IP, intraductal papilloma; IPC, intraductal papillary carcinoma.

− = absent to 5% of positive cells; + = >5–50%; ++ = >50%.

Chi-square test or Fisher's exact test, *P* > 0.05, compared with calponin, SMMHC or P63.
