# Supplementary material for: Novel Use for DOG1 in Discriminating Breast Invasive Carcinoma from Noninvasive Breast Lesions
Source: Dis Markers. 2016 Mar 2;2016:5628176. doi: 10.1155/2016/5628176 (PMC4793094; doi:10.1155/2016/5628176)
Supplement: Supplementary file 1 — In this study, we detected the expression of DOG1 in normal salivary gland. The result showed that it was DOG1 positive in the apical-luminal surface of serous acini but uniformly DOG1 negative in myoepithelial/basal cells, which was different from the performance of DOG1 in normal breast tissue. [file 5628176.f1.pdf]

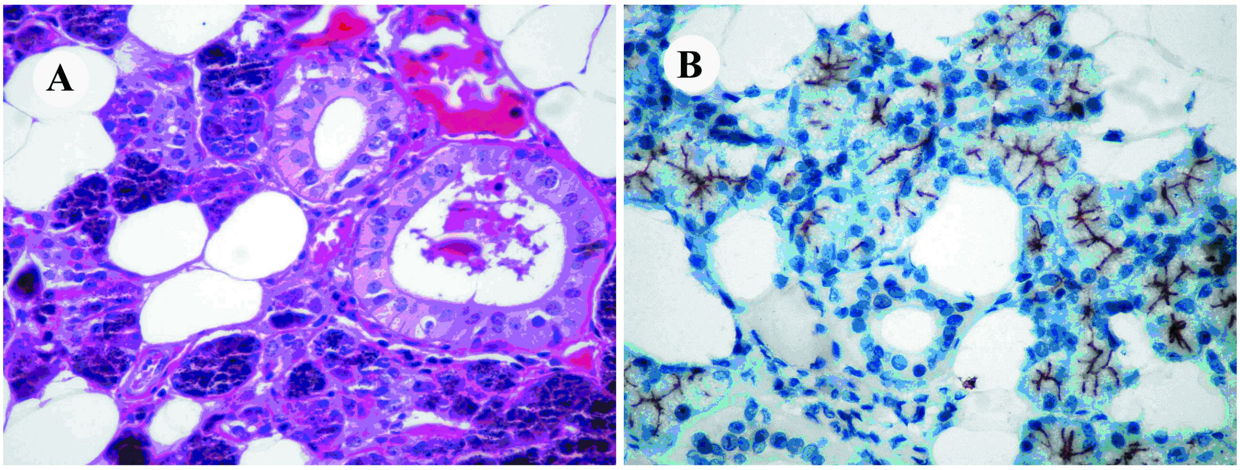

Supplementary Figure 1. DOG1 staining in normal salivary gland tissue (×400). A, Hematoxylin and eosin staining. B, DOG1 expression was positive in the serous acini of the parotid glands with an apical pattern of membranous staining; DOG1 expression was not found in the striated duct.
